# Rethinking return-to-office: the positive impact of remote work on well-being, connection, and employee retention

**DOI:** 10.3389/fpsyg.2026.1848857

**Published:** 2026-07-29

**Authors:** Alyssa M. Lezcano, Stephanie A. Zajac, Stefanie K. Johnson, Courtney L. Holladay

**Affiliations:** 1 Leadership Institute, University of Texas MD Anderson Cancer Center, Houston, TX, United States; 2 Leeds School of Business, University of Colorado Boulder, Boulder, CO, United States

**Keywords:** connection, hybrid work, remote work, turnover, well-being

## Abstract

**Introduction:**

As organizations reassess remote work policies in the post-pandemic era, questions remain about how flexible work arrangements impact employee well-being, connection, and turnover.

**Methods:**

Employing a time-lagged design with data from 7,704 employees across three work environments—remote (*n =* 1,869), hybrid (*n =* 2,099), and onsite (*n =* 3,736)—this study examines the psychological and organizational outcomes associated with work location.

**Results:**

Remote employees reported the highest well-being (M = 4.22), followed by hybrid (M = 4.12), and onsite workers (M = 3.89). ANOVA and post-hoc tests confirmed significant differences in well-being by work type, with remote workers reporting better outcomes. Similarly, positive workplace connection was mentioned at the highest rate among remote employees, though results were nonsignificant. Turnover rates examined 1 year post survey data collection were lowest for remote workers (7.8%) and highest for onsite employees (8.8%), though these differences were not statistically significant.

**Discussion:**

These findings challenge assumptions that remote work undermines connection and retention. Instead, they suggest that remote work can support well-being and that well-being, not proximity, is a key factor in employee retention. Implications for policy, leadership, and theoretical models of work are discussed.

## Introduction

While flexible work arrangements gained traction with the spread of internet access in the early 2000s, the COVID-19 pandemic forced a global and unprecedented shift to remote work, generating widespread interest in flexible work environments. Estimates indicate that as of early 2025, 13% of full-time workers were fully remote, while 26% maintained a hybrid schedule, and 61% worked entirely onsite (Barrero et al., 2025). Initially considered a means to improve work-life balance and maintain productivity, remote work was rapidly adopted and institutionalized by many organizations (Levy, 2020; Zeidner, 2020). Early narratives stated “it was here to stay,” emphasizing the potential benefits of remote and hybrid work arrangements for honoring employee well-being and expectations (Kossek et al., 2021; Robinson, 2022). To that end, employee expectations have shifted significantly, with many now actively expecting remote or hybrid work options as a standard component of employment (Buffer, 2023). However, recent reversals, such as organizational and governmental mandates for full returns to onsite work, have emerged despite limited empirical evidence assessing the consequences or benefits of such policies (Allen et al., 2025; Gibson et al., 2023; Hsu, 2025; Westfall, 2023). These abrupt shifts highlight unresolved questions about the psychological and social impacts of flexible work: specifically, questions remain on whether remote and hybrid work environments affect employee well-being, and to what extent remote work is inherently isolating.

Addressing these questions presents an important opportunity for research into the relational and psychological outcomes of varied work arrangements. Despite opposing forces supporting and restricting flexible work, much of the existing discourse—whether in support or opposition—lacks empirical rigor in evaluating its implications for employee and organizational outcomes. The present study seeks to contribute to this emerging literature by examining how different work arrangements (remote, hybrid, and onsite), relate to well-being, perceived connectedness, and turnover outcomes within an organizational context. By identifying relationships in these outcomes, we aim to provide a foundation for evidence-based workplace policy and decision-making.

## Background

Work arrangements are often viewed as operational or logistical choices, yet they have a considerable impact on the psychological mechanisms that shape how employees think and behave, ultimately influencing their satisfaction at work. Specifically, whether employees work onsite, remotely, or in a hybrid arrangement can shape their sense of autonomy, connectedness, and overall well-being—key mechanisms that can affect employee retention (Dysvik and Kuvaas, 2013; Feeley et al., 2008; Liu et al., 2011; Mossholder et al., 2005). This study draws on the Dual Pathway Model of Remote Work Intensity to examine the psychological and relational impacts of work arrangements and to understand how work environments may influence employee experience and behavior (Gajendran et al., 2024).

The ability to work remotely has become a key factor in attracting and retaining talent, with studies and surveys linking remote and hybrid work arrangements to higher job satisfaction, more flexibility and autonomy, and better work-life balance (Choudhury, 2025; Jamaludin and Kamal, 2023). Additionally, remote work supports inclusion and is seen as a way to expand opportunities and decrease discrimination for disabled workers, workers of color, women, caregivers, and people in the LGBTQ+ community (Allen et al., 2015; Amerikaner et al., 2023; Doering and Tilcsik, 2025; Santos et al., 2023). These benefits have made flexible work arrangements both a valued and sought after component of the employee experience and a strategic advantage for organizations in a competitive labor market. However, concerns have also been raised that remote workers may have a higher turnover risk due to reduced physical and social ties to the workplace and isolation (Van Zoonen and Sivunen, 2022). Importantly, these experiences are shaped by the technology that enables remote work, which Sonnentag et al. (2023) characterize as a double-edged sword for employee well-being. The authors argue that on one hand, technology-based communication can broaden social networks and support connection and well-being; on the other, heightened expectations for productivity, response immediacy, and constant availability may increase workload and strain, while reduced face-to-face interaction can undermine social support and contribute to loneliness.

Gajendran et al. (2024) propose a dual pathway model to capture these competing arguments and explain mixed outcomes associated with remote work. The authors present meta-analytic results that offer two opposing mechanisms through which remote work exerts its influence: perceived autonomy and perceived isolation. The pathway through autonomy suggests that remote work increases employee control over physical location and schedule, as well as decisions around how work is completed; this in turn supports well-being, the ability to focus, and a flexible work-life balance. How well remote workers communicate further influences key organizational and well-being outcomes such as performance and burnout (Shockley et al., 2021).

Media richness theory provides additional insight into this dynamic by explaining how the effectiveness of remote work depends on the richness of the communication media employees use (Daft and Lengel, 1986). Richer media—such as video conferencing or collaborative digital platforms—can better convey complex information and social cues, enabling employees to coordinate tasks more effectively and maintain a sense of control over their work processes (Aritz et al., 2018; Fuchs and Reichel, 2023). In this way, appropriate use of rich media may reinforce the autonomy pathway by enhancing clarity, reducing misunderstandings, and supporting more fluid communication despite physical distance. In an updated review of media richness theory, Ishii et al. (2019) cite that factors such as continuously evolving technology enriching previously “lean” media, availability of multiple channels with speed and convenience, and new features that promote connectivity, have all transformed effective communication.

In contrast, the pathway through isolation suggests that physical separation from colleagues and supervisors reduces access to support and information, as well as erodes one’s social connection. Research on remote work indicates that social isolation is negatively related to satisfaction with remote work arrangements (Toscano and Zappalà, 2020), highlighting the relational costs -- fear of missing out and lack of belonging -- that may accompany reduced proximity (Afota et al., 2024; Reimann et al., 2025). More broadly, Van Zoonen and Sivunen (2022) identify isolation as a focal challenge for organizations operating with dispersed workforces. The authors’ work links feelings of isolation to psychological distress, including unhappiness and negative affect, underscoring why isolation represents a critical concern in an era of increasing attention to employee health and well-being.

Media richness theory helps explain this effect, as less rich communication channels may fail to convey emotional and social cues that sustain interpersonal relationships and social cohesion. The authors argue that electronically mediated interactions may not substitute for the spontaneous social interactions (e.g., water cooler conversations, employee celebrations) that occur in the physical workplace. Thus, when remote work relies heavily on lean communication media, employees may experience heightened perceptions of isolation, underscoring the importance of selecting communication modes that match the complexity and social demands of workplace interactions.

The Dual Pathway Model offers a valuable framework from which to understand how work arrangements might lead to either beneficial or detrimental outcomes. Hybrid work may offer a potential balance by providing some autonomy while maintaining regular opportunities for connection. However, with only a few notable exceptions (e.g., Singh and Sant, 2023), empirical work is lacking, and the effect of work arrangement on turnover, as well as the role of underlying psychological mechanisms, remain empirical questions.

Building on this background, it is essential to examine the specific outcomes that are most relevant to understanding the broader impact of remote work. In particular, this study focuses on three primary domains of interest: well-being, connection, and retention. These outcomes were selected based on their relevance to both individual-level experiences and broader organizational goals, and they serve as key indicators for exploring the effectiveness and sustainability of current practices. The following sections examine each outcome in detail.

### Well-being

A core outcome of interest in this study is employee well-being. Danna and Griffin (1999) conceptualize well-being as a multidimensional construct, distinguishing between job-related satisfactions and broader life satisfactions. Charalampous et al. (2019) present a framework that further delineates well-being at work to include affective, cognitive, social, professional, and psychosomatic dimensions. Their research on remote work suggests that flexible arrangements are often associated with positive affective and professional outcomes, such as higher job satisfaction and perceptions of inclusion, alongside potential social and professional-related risks, including reduced social support and concerns about advancement. Notably, much of this literature predates the widespread, sustained adoption of remote work, leaving open questions about how current work arrangements shape employee well-being. Building on this foundation, the present study draws on the Dual Pathway perspective to examine how features of different work arrangements may simultaneously enhance well-being through increased autonomy while undermining it through experiences of isolation.

While there is widespread interest in how flexible work arrangements affect well-being, evidence suggests that one key mechanism operates through autonomy. Autonomy—defined as a sense of willingness and freedom over one’s actions—has been identified as a basic and universal human need (Ryan and Deci, 2019). Across a range of occupational contexts, autonomy has been shown to exert a direct positive effect on employee well-being, including knowledge-intensive, client-facing, production, transportation, sales, marketing roles, and gig work (Clausen et al., 2022; Wan et al., 2024). Flexible and remote work arrangements may enhance autonomy, and therefore well-being, by increasing employees’ discretion over when, where, and how work is accomplished (George et al., 2025). Alternatively, autonomy may function as a buffer against stressors inherent in traditional on-site work, such as long or unpredictable commuting demands (Emre and De Spiegeleare, 2021). Differences in well-being across work arrangements, therefore, may be due in part to an employee’s experience of autonomy as a core psychological need.

In contrast, a second pathway emphasizes the potential costs of flexible work arrangements through experiences of isolation, which have been linked to poorer well-being (George et al., 2022). O’Hare et al. (2024) conducted research during large-scale shifts to remote work and found that workplace isolation and loneliness are associated with lower levels of well-being, particularly when employees perceive diminished access to colleagues and organizational support networks. The authors argue that reduced informal interaction and the task-focused nature of virtual meetings may limit opportunities for social connection, leaving employees’ psychological need for belonging unmet. Similarly, Becker et al. (2022) find that work-related loneliness is negatively associated with well-being, which subsequently leads to adverse health outcomes, including depression and insomnia. However, much of this evidence emerged during periods when remote work was rapidly and involuntarily adopted, often without adequate technological infrastructure, norms, or intentional practices to support connection. As organizations have since developed more advanced tools and approaches for remote and hybrid work, the extent to which isolation continues to undermine well-being remains unanswered.

This study explores whether well-being levels differ meaningfully across remote, hybrid, and onsite work environments. Understanding how work structure relates to well-being is vital, as organizations increasingly emphasize employee experience and mental health as strategic priorities (Greenwood and Anas, 2021). If certain arrangements systematically support or hinder well-being, this could have implications not only for individual health, but also for absenteeism, turnover, and long-term organizational effectiveness (Cooper and Dewe, 2008). Hybrid work may offer an opportunity to balance autonomy and connection, but it also introduces its own complexities, such as difficulty coordinating work schedules, tasks, and timelines (Wigert and White, 2022). By investigating these possibilities, this study seeks to uncover which work arrangement is most conducive to promoting well-being.

### Positive connection

One important area of exploration in this study is how different work arrangements may affect employees’ sense of connection with colleagues and their broader organization. Connection impacts various aspects of work including teamwork, mental health, and even employee engagement (Mittal et al., 2025). As more organizations experiment with remote and hybrid models, it remains unclear how these arrangements influence connection. In this study, we use the term positive connection to describe employees’ perceptions of cohesion, care, and collaboration within the organization. While related constructs (e.g., belonging, connectedness) emphasize interpersonal closeness, positive connection focuses on the positive, relational qualities that characterize a supportive workplace culture.

Although research directly examining the relationship between autonomy and employees’ sense of connection to colleagues and the organization is limited, related evidence from the work engagement literature offers a useful foundation. Prior studies consistently demonstrate that autonomy is positively associated with work engagement, a motivational state characterized by vigor, dedication, and absorption (Bakker et al., 2008). At the team level, autonomy has been shown to have small but statistically significant effects on engagement, with this relationship mediated by the quality of relationships between team members and leaders (Palumbo, 2021). These findings suggest that autonomy may foster richer social exchanges and a more supportive organizational climate, both of which are inherently relational in nature. Similarly, De Spiegelaere et al. (2016) find that work method autonomy—defined as decision latitude over processes, methods, and ways of working—enhances engagement by enabling employees to become more deeply involved in and motivated by their work. Extending this logic, autonomy may not only energize individuals’ task involvement but also strengthen their sense of positive connection by increasing opportunities for meaningful interaction and discretionary collaboration. In this way, autonomy may indirectly support positive connection in the workplace through its beneficial effects on engagement and relational quality.

A perceived lack of social support or relational embeddedness in remote work might lead to feelings of isolation and a reduced sense of belonging. Qualitative work has highlighted that a recurring challenge remote employees experience is the erosion of collegial ties, such that the spontaneity and frequency of casual social interactions and informal social support in a remote setting is reduced compared to working onsite (Yalçınyiǧit, 2026). This relationship is particularly consequential given evidence that social connection is a fundamental determinant of health, with lack of social connection identified as a significant risk factor for poor physical health and adverse health outcomes (Holt-Lunstad, 2021). Indeed, support from peers has been shown to relate positively to perceptions of well-being for remote workers (Capone et al., 2024). In remote work contexts, isolation has been shown to undermine relational processes that support connection. For example, Chinyuku and Qutieshat (2025) examine the impact of remote work on cohesion, a closely related construct, and argue that isolation can disrupt communication patterns, hinder rapport-building, and weaken emotional ties among coworkers. Related work on teleworking leaders suggests that physical and psychological distance may also shape how employees interpret and relate to others at work; specifically, extensive leader teleworking has been linked to lower follower trust, in part because followers form more abstract and less nuanced understandings of their leaders (Golden and Ford, 2025). This effect appears particularly pronounced in contexts characterized by high levels of leader monitoring, where reduced autonomy further undermines relational trust. Importantly, Chinyuku and Qutieshat (2025) emphasize that these effects are not inevitable, and deliberate communication strategies and intentional approaches to interaction may help mitigate the relational costs of isolation. Together, this work suggests that while isolation poses a meaningful threat to positive connection in the workplace, organizational practices and intentional approaches to virtual communication may play a critical role in determining whether remote work arrangements negatively impact employees’ sense of connection.

Hybrid models might offer a balance—allowing for some in-person contact without sacrificing flexibility—but the effectiveness of these models in fostering strong, positive connection remains an open question. By examining this dimension empirically, the study contributes to understanding whether certain work arrangements better support employees’ need for meaningful connection at work.

### Retention

Employee retention is a particularly consequential outcome to examine in the context of evolving work arrangements. As organizations adapt to post-pandemic workforce expectations, the ability to retain talent has become increasingly tied to the quality and flexibility of work experiences. There is growing interest in whether offering remote or hybrid work can reduce turnover, yet it remains unclear which arrangements are most effective at fostering long-term commitment. Some suggest that flexible work enhances retention by aligning with employees’ personal needs and preferences, while others argue that it may lead to lower performance and difficulty onboarding new hires (Cappelli and Nehmeh, 2025; Singh and Sant, 2023). This study seeks to explore how work arrangements might influence whether employees stay or leave.

Retention is a critical metric for organizations navigating an increasingly mobile and competitive labor market. High turnover incurs substantial costs in the form of recruitment, training, and lost productivity—and may be the result of deeper issues related to organizational commitment and job satisfaction (Rubenstein et al., 2018). By exploring whether certain work arrangements are associated with stronger retention outcomes, this study addresses both practical and theoretical questions. Specifically, we investigate whether employees working in remote, hybrid, or onsite environments are more or less likely to stay, and how psychological mechanisms like connection and well-being may factor into those decisions.

Together, well-being, connection, and retention represent critical outcomes for understanding the individual and organizational impacts of work environment. To investigate how these outcomes are shaped by remote, hybrid or onsite work, we pose the following research questions.

*Research Question 1*: What is the relationship between work environment (remote, hybrid, onsite) and well-being?

*Research Question 2*: What is the relationship between work environment (remote, hybrid, onsite) and connection?

*Research Question 3*: Does work environment (remote, hybrid, onsite) affect turnover through well-being and positive connection?

## Methods

### Data

Our study employs a time-lagged design from a larger sample of employees at a large healthcare organization in the Southwestern United States. These employees participated in an engagement survey in 2023 and were designated as permanent /regular employees prior to the time of the survey (temporary employees, those working per diem, and educational appointees were excluded from the sample). For the larger sample, of the 23,316 employees invited, 20,856 responded, resulting in an 89% response rate. For our study sample, only those respondents who answered the open-ended question describing the company culture were included, described further in our connection measure below. Descriptive analyses indicated that participants excluded due to missing qualitative responses did not meaningfully differ from those retained in the final sample in age, tenure, gender, work environment, or turnover. Outside of these constraints, employees from all departments were included to allow for the largest sample of remote, hybrid (working a mix of remote and onsite), and fully onsite employees. Thus, the final sample for this study included 7,704 employees, the majority of whom were female (68%) with a mean age of 47 years. In terms of race and ethnicity, the sample was 27% Asian, 18% Hispanic/Latino, 30% White, 22% Black/African American, and 3% other/multiple ethnicities. The full sample of 7,704 employees can be split into three subsamples: 1,869 remote employees, 2,099 hybrid employees, and 3,736 employees who work fully onsite. One year following the survey collection, turnover data was collected to complete the study design.

### Measures

*Work environment:* managers assign every employee to a work environment classification within the human resources information system based on their scheduled work arrangement. Managers use the following guidelines for classification: employees working fully remote with zero days onsite were considered remote; employees working a mix of onsite and remote work schedules (typically 2–3 days per week onsite) were considered hybrid employees, and those working fully in-person with zero days remote were considered onsite. We coded this as a categorical variable, where 1 = Remote, 2 = Hybrid and 3 = Onsite.

*Well-being:* four items were used to capture employees’ overall perceptions of well-being: “[My organization] cares about my health and well-being”; “I feel energized by my work”; “I feel supported by my manger in making decisions about my health and well-being”; “I am able to find life balance.” Each item was answered on a Likert-type scale ranging from 1 (“Strongly Disagree”) to 5 (“Strongly Agree”). While these items were not pulled from an existing well-being measure, the scale exhibited good reliability with a standardized Cronbach’s *α* of 0.84. Additionally, in a confirmatory factor analysis of the 4-item measure, all the items loaded onto the higher-order factor (between 0.69–0.86) and the best fitting model was the model with all four items loading onto the single factor (CFI = 0.99; TLI = 0.99; RMSEA = 0.049; SRMR = 0.02).

*Positive connection:* our measure of positive connection originated from employees’ qualitative responses to an open-ended item that asked, “What 3–5 words would you use to describe [our organization’s] culture?” Responses were only coded if they included at least 3 descriptive words. First, three of the authors compiled a list of all of the words employees provided and independently rated each word as to whether or not it was an indicator of positive connection in the workplace. Consistent with our conceptualization of positive connection, words reflecting inclusion, unity, care, and collaboration were identified and included. The authors then met as a group to discuss any words that did not have complete agreement and made decisions about whether to include them in the subsequent analyses. After this iterative process, all the words coded as indicating positive connection at work were created into a dictionary which was then imported into LIWC-22 (Boyd et al., 2022), a software used for analyzing word use (see Table 1 for dictionary). LIWC-22 provided the percent to which each respondent’s qualitative answer represented positive connection per the dictionary the authors created. Given that employees provided varying numbers of words to answer the question (minimum of 3, maximum of 5), we coded positive connection as a dichotomous variable. If the response included any positive connection words, they were coded as 1; responses that had zero positive connection words were coded as 0.

**Table 1:** Positive connection – dictionary.

Words indication positive connection		
Accept	Employee-friendly	United
Access	Employee-supportive	Unity
Accommodating	Encompassing	Welcome
Affable	Engage	Welcoming
All backgrounds	Ethnic	Work together
All Faiths	Familial	Working together
All-inclusive	Family	
Amazing Colleagues	Friend	
Amazing Leadership	Friendship	
Amiable	Generational	
Amicable	Good teamwork	
At-home	Great team	
Belonging	Great working relations	
Bond	Helpful coworkers	
Buddy-system	Including	
Camaraderie	Inclusion	
Care	Inclusive	
Caring	Melting pot	
Caring staff	Mentor	
Caring/Compassion	Military friendly	
Caring/Supportive	Multi-cultural	
Close	multi-generational	
Close-knit	Multinational	
Cohesion	Network	
Cohesive	Nice colleagues	
Collaborate	Non-biased	
Collective	Non-discriminating	
Collegial	Non-Discriminatory	
Common goal	One family	
Common purpose	One team	
Communal	People(and team)-oriented	
Community	People-friendly	
Companion	Relational	
Comradery	Relationship	
Connect	Team	
DEI	Together	
Diverse	Unbiased	
Diversity	Unite	

*Turnover:* turnover was measured 1 year after the engagement survey and was coded as a dichotomous variable where 1 = Turned Over and 0 = Active.

*Tenure:* years of tenure were measured by calculating the difference between the employees’ start date at the organization and the time of survey collection.

## Results

Correlations between our variables of interest for our sample of 7,704 employees can be found in Table 2. Analyses were conducted using the psych and lavaan packages in R studio (Revelle, 2025; Rosseel, 2012).

**Table 2:** Means, standard deviations, and correlations with confidence intervals.

Variable	*M*	*SD*	1	2	3	4	5
1. Work Environment	2.24	0.82					
2. Well-Being	4.04	0.82	−0.17** [−0.19, −0.15]				
3. Positive Connection	0.57	0.49	−0.05** [−0.07, −0.03]	0.14** [0.12, 0.17]			
4. Turnover	0.08	0.28	0.01 [−0.01, 0.04]	−0.08** [−0.10, −0.06]	−0.02* [−0.05, −0.00]		
5. Age (Years)	47.62	10.85	−0.06** [−0.08, −0.03]	0.12** [0.10, 0.14]	0.01 [−0.01, 0.03]	0.03* [0.01, 0.05]	
6. Tenure (Years)	12.80	8.03	−0.07** [−0.10, −0.05]	0.04** [0.02, 0.07]	0.02 [−0.01, 0.04]	−0.05** [−0.07, −0.02]	0.51** [0.49, 0.53]

Work Environment: Remote = 1, Hybrid = 2, Onsite = 3. Positive Connection: 1 = Positive Connection, 0 = Zero Positive Connection Words. Turnover: 1 = Turned Over, 0 = Active Employee. M and SD are used to represent mean and standard deviation, respectively. Values in square brackets indicate the 95% confidence interval for each correlation. *p < 0.05. **p < 0.01.

Our first research question investigated whether and how work environments affect employee well-being. To test this, we first examined the direct relationships between employee work environments (i.e., remote, hybrid, onsite) and our measure of well-being. In terms of descriptive statistics, well-being scores decreased from 4.22 to 4.12 when comparing remote and hybrid samples, with the onsite sample having the lowest average score of 3.89. A one-way analysis of variance (ANOVA) revealed significant differences in well-being across work environments [*F*(2, 7,648) = 117.9, *p <* 0.001]. Again, well-being was highest for remote employees (M = 4.22, 95% CI [4.19, 4.26]), followed by hybrid (M = 4.12, 95% CI [4.09, 4.16]), and onsite employees (M = 3.9, 95% CI [3.87, 3.92]). A post-hoc Tukey test revealed that remote employees reported significantly higher well-being than both hybrid (mean difference = 0.10, *p =* 0.0003, 95% CI [0.04, 0.16]) and onsite workers (mean difference = 0.33, *p <* 0.001, 95% CI [0.27, 0.38]). Hybrid employees also reported significantly higher well-being than onsite workers (mean difference = 0.23, *p <* 0.001, 95% CI [0.23, 0.28]). Regression analyses, weighted according to subsample N size, also point to significant differences in well-being according to work environment, with onsite employees reporting the lowest and remote employees reporting the highest well-being (see Table 3). These differences were aligned with the pattern observed in mean scores, indicating that employees in remote work environments reported the highest well-being on average, followed by hybrid and then onsite workers.

**Table 3:** Work environment regression results.

Dependent Variable	Predictor	*B*	*SE*	95% CI [LL, UL]	*p*
Well-Being	(Intercept - Hybrid)	4.12	0.02	[4.09, 4.15]	<0.001
	Onsite	−0.23	0.02	[−0.27, −0.18]	<0.001
	Remote	0.10	0.02	[0.05, 0.14]	<0.001
Positive Connection	(Intercept - Hybrid)	0.34	2.02		0.86
	Onsite	−0.14	2.85		0.96
	Remote	0.11	2.88		0.96
Turnover	(Intercept - Hybrid)	−2.39	3.62		0.51
	Onsite	0.06	5.05		0.99
	Work Environment	−0.06	5.18		0.99

Work Environment: Remote = 1, Hybrid = 2, Onsite = 3. Positive Connection: 1 = Positive Connection, 0 = Zero Positive Connection Words. Turnover: 1 = Turned Over, 0 = Active Employee. LL and UL indicate the lower and upper limits of a confidence interval, respectively. Regressions were weighted according to work environment sample size. Positive connection and Turnover analyses were run using binomial logistic regression.

Our second research question concerned the extent to which remote work may be inherently isolating. To better understand how an employee’s work environment relates to perceptions of positive connection, we first examined the correlations between work environment categories and whether employees’ qualitative responses included words indicating positive connection at work. Interestingly, positive connection also had mean differences according to work environment. Specifically, the remote sample had the highest average mention of perceived positive connection (M = 0.61), while the hybrid (M = 0.58) and onsite (M = 0.55) samples had slightly lower mean values. To test whether the distribution of our binary outcome, connection, differed across the three samples, we used Pearson’s Chi-Squared test; this method is appropriate for comparing a trichotomous independent variable and a binary dependent variable. The Pearson’s Chi-squared test revealed a significant association between work environment and mention of perceived positive connection [*χ*^2^ (2) = 21.30, *p <* 0.001]. Using binomial logistic regression, appropriate for a dependent variable with two categories, we observed a similar pattern to the well-being outcome. This regression analysis, found in Table 3, also suggests that the average amount of mention of perceived positive connection is lowest for onsite workers and highest for those working remotely, although these estimates are nonsignificant when weighted according to work environment sample size. Overall, our data suggests that while the differences are minimal, remote employees in our sample mention perceived positive connection more, while onsite employees mention perceived positive connection the least.

In our third research question, we examined the relation between work arrangement and a more distal organizational outcome – turnover – through well-being and positive connection. While the overall rate of turnover in our sample was quite low (~8%), the average amount of turnover showed small differences between each work environment. Remote work (M = 0.078), hybrid work (M = 0.083), and onsite work (M = 0.088) show increasing rates of turnover as work transitions to a more in-person environment, although these differences are small given the low rate of total turnover. Before testing the indirect effects, we note that well-being was negatively related to turnover, but positive connection was unrelated to turnover (see Tables 4, 5). Thus, we tested the indirect effect of work environment on turnover through well-being, following a regression-based approach consistent with Hayes’ PROCESS Model framework (Hayes, 2022). Specifically, the mediation procedure involved two steps. We first fit a linear model predicting the mediator (i.e., work environment predicting well-being) to estimate the effect of work environment group membership on well-being. Next, we ran a logistic regression model predicting the outcome (i.e., well-being predicting turnover) to estimate the effect of both group membership and well-being on turnover. To facilitate interpretation and reduce multicollinearity, the well-being variable was mean-centered prior to these analyses. Work environment was dummy-coded, with the onsite work group as the referent. The mediator model again showed that work environment group was a significant predictor of well-being [*F*(2, 7,648) = [117.9], *p <* 0.001], with average well-being highest for remote employees and lowest for onsite employees. In the outcome model, which included both well-being and work environment as predictors, only well-being had a significant, negative effect on turnover (*B* = −0.33, *p <* 0.01, 95% CI [−0.42, −0.23]); this translates to an odds ratio of 0.71, suggesting that greater well-being decreased the odds of turnover. The full results for both models can be found in Table 6.

**Table 4:** Comparisons of well-being and turnover regressions.

Work environment	Predictor	*B*	*SE*	95% CI [LL, UL]	*p*
Remote	(Intercept)	−0.89	0.44	[−1.79, −0.05]	<0.05
	Well-Being	−0.38	0.11	[−0.59, −0.17]	<0.001
Hybrid	(Intercept)	−0.63	0.36	[−1.35, 0.06]	>0.05
	Well-Being	−0.44	0.08	[−0.62, −0.26]	<0.001
Onsite	(Intercept)	−1.38	0.24	[−1.86, −0.92]	<0.001
	Well-Being	−0.25	0.06	[−0.37, −0.13]	<0.001

Turnover: 1 = Turned Over, 0 = Active Employee. Remote *N =* 1869; Hybrid *N =* 2099; Onsite *N =* 3,736. LL and UL indicate the lower and upper limits of confidence intervals, respectively. Analyses were run using binary logistic regression.

**Table 5:** Comparisons of positive connection and turnover regressions.

Work environment	Predictor	*B*	*SE*	95% CI [LL, UL]	*p*
Remote	(Intercept)	−2.32	0.13	[−2.58, −2.07]	<0.001
	Positive Connection	−0.24	0.17	[−0.58, 0.09]	>0.05
Hybrid	(Intercept)	−2.31	0.12	[−2.54, −2.08]	<0.001
	Positive Connection	−0.16	0.16	[−0.47, 0.15]	>0.05
Onsite	(Intercept)	−2.26	0.08	[−2.43, −2.10]	<0.001
	Positive Connection	−0.14	0.12	[−0.36, 0.09]	>0.05

Turnover: 1 = Turned Over, 0 = Active Employee. Remote *N =* 1869; Hybrid *N =* 2099; Onsite *N =* 3,736. LL and UL indicate the lower and upper limits of confidence intervals, respectively. Analyses were run using binary logistic regression.

**Table 6:** Process model regressions.

Model	Predictor	*B*	*SE*	95% CI [LL, UL]	*p*
Mediator (Work Environment Predicting Well-Being)	(Intercept - Onsite)	−0.14	0.01	[−0.16, −0.11]	<0.001
	Hybrid	0.23	0.02	[0.18, 0.27]	<0.001
	Remote	0.33	0.02	[0.28, 0.37]	<0.001
Outcome (Work Environment and Well-Being Predicting Turnover)	(Intercept - Onsite)	−2.42	0.06	[−2.54, −2.30]	<0.001
	Hybrid	0.01	0.09	[−0.19, 0.20]	0.92
	Remote	−0.01	0.11	[−0.22, 0.19]	0.93
	Well-Being	−0.33	0.05	[−0.42, −0.23]	<0.001

Turnover: 1 = Turned Over, 0 = Active Employee. Work Environment is dummy-coded, with onsite work as the referent. Well-Being is mean-centered. LL and UL indicate the lower and upper limits of confidence intervals, respectively. Analyses were run using a linear model (Mediator Model) and binary logistic regression (Outcome Model).

Indirect effects were estimated using nonparametric bootstrapping with 1,000 resamples to generate 95% confidence intervals. Mediation would be considered significant if the confidence interval of the indirect effect did not include zero. Bootstrapped indirect effects revealed that the average causal mediation effects, or the indirect effects of work environment via well-being, were significant across all sample comparisons (Table 7). In each of these models, these indirect effects were negative and had 95% confidence intervals that did not include zero. No direct effects were significant, suggesting that the relationship between work environment and turnover may be fully mediated by well-being. Overall, our results confirm that there is a statistically significant indirect effect of work environment on turnover via well-being and that this mediated effect, while minimal, may contribute to a reduction in the likelihood of employee turnover.

**Table 7:** Pairwise mediation analysis comparing results by work environment group.

Model	Effect type	Estimate (B)	95% CI [LL, UL]	*p*
Hybrid vs. Onsite (Control)	ACME (Indirect)	−0.01	[−0.01–0.00]	<0.001
	ADE (Direct)	0.00	[−0.01, 0.01]	0.96
	Total Effect	−0.00	[−0.02, 0.01]	0.52
	Proportion Mediated	1.16	[−6.70, 7.91]	0.52
Remote vs. Onsite (Control)	ACME (Indirect)	−0.01	[−0.01, −0.01]	<0.001
	ADE (Direct)	−0.00	[−0.02, 0.01]	0.90
	Total Effect	−0.01	[−0.02, 0.01]	0.23
	Proportion Mediated	−0.92	[−6.07, 9.26]	0.23
Remote vs. Hybrid (Control)	ACME (Indirect)	−0.002	[−0.00, −0.00]	<0.001
	ADE (Direct)	−0.00	[−0.02, 0.01]	0.88
	Total Effect	−0.00	[−0.02, 0.01]	0.69
	Proportion Mediated	0.63	[−4.09, 3.31]	0.69

Turnover: 1 = Turned Over, 0 = Active Employee. Work Environment is dummy-coded, with onsite work as the referent. Well-Being is mean-centered. LL and UL indicate the lower and upper limits of confidence intervals, respectively. All estimates are unstandardized, and confidence intervals are bias-corrected and bootstrapped (1,000 resamples).

In addition to the PROCESS model, we used structural equation modeling to test an indirect path model in which well-being and positive connection mediated the relationship between work environment and turnover. Ultimately, the model did not have good fit but did show similar results to the PROCESS model; specifically, well-being was again the only significant mediator.

### *Post-hoc* analyses

In addition to our examination of the variables addressed in our research questions, we also investigated employee age, tenure, and gender as potential explanatory variables of the relationships between work environment and both connection and well-being. Firstly, age is a demographic factor that may influence well-being, connection and relationship-formation at work, as well as how and when employees leave the organization. Employees in the full sample ranged from 22 to 85 years old (M = 47.6; SD = 10.8). Average age across work environment subsamples were relatively similar (Remote: M = 48.5; Hybrid: M = 47.8; Onsite: M = 47.1). ANOVA revealed that there were significant differences in age according to work environment [*F*(2, 7,701) = 12.08, *p <* 0.001], with estimated means highest for remote employees (M = 48.5, 95% CI [48.0, 49.0]), followed by hybrid (M = 47.8, 95% CI [47.3, 48.3]), and onsite employees (M = 47.1, 95% CI [46.7, 47.4]). Tukey multiple comparisons of means also revealed that onsite workers were significantly younger than both remote workers (mean difference = 1.48 years, *p <* 0.001, 95% CI [1.48, 2.20]) and hybrid workers (mean differences = −0.75 years, *p =* 0.03, 95% CI [−1.44, −0.06]). The difference in age between remote and hybrid workers was not statistically significant and estimated confidence intervals included zero (mean difference = 0.73 years, *p =* 0.08, 95% CI [0.07, 1.54]). Regression analyses (Table 8), further suggest that age was significantly related to work environment, such that onsite workers had the lowest average age (*B* = −0.75, *p <* 0.05, 95% CI [−1.33, −0.16]) and remote workers had the highest average age (*B* = 0.73, *p <* 0.05, 95% CI [0.14, 1.32]). This, along with the differences in subsample means, implies that employees may be slightly younger in onsite positions compared to remote and hybrid positions. Regarding additional related outcomes, employee age was significantly and positively correlated with well-being (0.12, *p <* 0.01, 95% CI [0.10, 0.14]) and turnover (0.03, *p <* 0.05, 95% CI [0.01, 0.05]). However, weighted regression coefficients between age and well-being, connection, and turnover were near zero and nonsignificant.

**Table 8:** Tenure and age regression results.

Dependent variable	Predictor	B	*SE*	95% CI [LL, UL]	*p*
Age analyses					
Age	(Intercept - Hybrid)	47.81	0.21	[47.39, 48.22]	<0.001
	Onsite	−0.75	0.30	[−1.33, −0.16]	0.01
	Remote	0.73	0.30	[0.14, 1.32]	0.01
Well-Being	(Intercept)	3.69	0.04	[3.61, 3.77]	<0.001
	Age	0.01	0.00	[0.01, 0.01]	<0.001
Positive Connection	(Intercept)	0.22	5.31		0.97
	Age	0.00	0.11		0.98
Turnover	(Intercept)	−2.93	9.67		0.76
	Age	0.01	0.19		0.95
Tenure analyses					
Tenure	(Intercept - Hybrid)	13.03	0.16	[12.71, 13.33]	<0.001
	Onsite	−0.79	0.22	[−1.24, −0.36]	<0.001
	Remote	0.65	0.22	[0.21, 1.08]	<0.001
Well-Being	(Intercept)	4.02	0.02	[3.98, 4.06]	<0.001
	Tenure	0.00	0.00	[0.00, 0.01]	<0.001
Positive Connection	(Intercept)	0.27	2.22		0.90
	Tenure	0.00	0.15		0.97
Turnover	(Intercept)	−2.14	3.84		0.57
	Tenure	−0.02	0.27		0.94

Work Environment: Remote = 1, Hybrid = 2, Onsite = 3. Positive Connection: 1 = Positive Connection, 0 = Zero Positive Connection Words. Turnover: 1 = Turned Over, 0 = Active Employee. LL and UL indicate the lower and upper limits of a confidence interval, respectively. Regressions were weighted according to work environment sample size. Positive connection and Turnover analyses were run using binomial logistic regression.

The amount of time employees have worked at the organization may also have an impact on our variables of interest. On average, employees in our sample had been at the organization for 12.8 years (SD = 8.03) and ranged from less than 1 year to 47 years. A one-way ANOVA indicated significant differences in employee tenure across work environments [*F*(2, 7,695) = 21.47, *p <* 0.001]. Average tenure was highest for remote employees (M = 13.7, 95% CI [13.3, 14.0]), while hybrid (M = 13.0, 95% CI [12.7, 13.4]) and onsite (M = 12.2, 95% CI = 12.0, 12.5]) employees had lower average tenure. Tukey’s post-hoc tests revealed that remote employees had significantly longer tenure than both hybrid (mean difference = 0.65 years, *p =* 0.03, 95% CI = [0.05, 1.24]) and onsite workers (mean difference = 1.44 years, *p <* 0.001, 95% CI [0.91, 1.97]). Hybrid employees also had significantly longer tenure than onsite employees (mean difference = 0.80 years, *p =* 0.001, 95% CI [0.28, 1.31]). Weighted regression results also show significant differences across work environment grouping, such that onsite workers have the shortest average tenure (*B* = −0.79, *p <* 0.001, 95% CI [−1.24, −0.36]) and remote workers have the longest average tenure (*B* = 0.65, *p <* 0.01, 95% CI [0.21, 1.08]). Tenure had significant correlations with well-being (0.04, *p <* 0.01, 95% CI [0.02, 0.07]) and turnover (−0.05, *p <* 0.01, 95% CI [−0.07, −0.02]), but did not have meaningful relationships with well-being, positive connection, or turnover when run as regressions.

Finally, we also tested gender as a potential moderator of the relationships between work environment and our two mediators. Social role theory (Eagly and Wood, 2012) and gender stereotypes would suggest that women may place greater value on communality than men and thus may have differing experiences connecting at work depending on the level of virtuality of the work environment. We tested this potential boundary condition for both well-being and positive connection. While there were mean differences according to gender (females reported significantly more positive connection than males in all work environments), interaction terms for gender moderating the proposed relationships were nonsignificant. For the sake of parsimony and clarity, we retain the primary findings as the results.

## Discussion

This study directly responds to growing calls for empirical insight into the psychological and social consequences of flexible work environments, which have become central to both policy debates and organizational strategies in the post-COVID era. The rigor of our design enhances the strength of these contributions. By having defined employee work environments at the outset, then administering survey measures, and finally linking these data to actual turnover 1 year later, this study affords an unusually robust test of the relationships among work arrangement, employee experiences, and retention outcomes. This approach increases confidence that the observed patterns reflect meaningful associations rather than transient perceptions or measurement artifacts. In addressing our first research question—how remote and hybrid work environments affect employee well-being—we find that remote work is significantly associated with higher reported well-being. This gradient, with well-being highest among fully remote workers and lowest among those onsite, supports early pandemic-era assumptions regarding remote work associations with psychological health.

Our second research question explored whether remote work is inherently isolating, as often feared. No statistically significant differences were observed. However, remote employees in our sample mentioned perceived positive connection at a slightly greater rate than their hybrid and onsite peers, which may suggest that remote work is not necessarily associated with lower levels of workplace cohesion and collaboration. These findings may point toward a more nuanced understanding of work environment and connection—one that may be shaped by intentional communication practices, rather than mere proximity. Indeed, it may be communication quantity and quality rather than whether someone is remote or in person that impacts the effectiveness of remote teams (Shockley, et al., 2021). Because all data were collected within a single organization, we inherently controlled for organizational culture, norms, and systems, allowing us to more precisely isolate the relationship between work arrangement and perceived connection. Our results are also in keeping with qualitative findings that suggest all employees may experience similar perceptions of isolation once a certain number of them work remotely, further distributing employees from one another (Rockmann and Pratt, 2015). Thus, our findings offer an evidence-based counterpoint to recent reversals in remote work policies, which often rely on untested assumptions about social cohesion.

Regarding our third research question related to turnover, our data indicated minimal variation in actual turnover rates by work environment, with slightly higher turnover observed among onsite workers. However, these differences were not statistically significant, suggesting that work environment alone may not be a strong direct predictor of turnover intentions or behavior in this context. Importantly, well-being emerged as a consistent and significant negative predictor of turnover across all groups. Conversely, although positive connection showed a negative relationship with turnover, this association was not statistically significant, indicating that connection may play a more complex or indirect role in retention.

### Limitations

Several limitations must be acknowledged when interpreting these findings. First, while collecting data from a single organizational site is advantageous because it allows tighter control over organizational variables and shared norms, relying on one site also limits how far the study’s findings can be generalized. This constraint may mean the results do not fully capture the diversity of practices, cultures, or conditions present in other organizations, reducing the broader applicability of the conclusions. Further, the unique culture of a large healthcare setting—including mission-driven work, complex staffing structures, and regulatory constraints—may influence how remote or hybrid work is experienced. Thus, results may differ in industries with different work norms, autonomy levels, or technological infrastructures.

Second, our measurement of perceived positive connection relied on the presence of related keywords in employee responses, which may not fully capture the depth or quality of workplace relationships. While this approach offers a scalable and objective indicator, it may miss more subtle or complex relational dynamics, such as emotional closeness, trust, or frequency of interaction. Moreover, positive connection may be context-dependent, and its expression can vary across cultural or organizational norms, limiting the generalizability of our findings. Additionally, the positive connection variable was derived from a researcher-created dictionary and subsequently dichotomized, which may have reduced sensitivity to variation in employees’ experiences and obscured potentially meaningful nuances in workplace relationships.

Two additional limitations warrant note. Work arrangement in a healthcare context is often intertwined with job function; remote roles tend to be concentrated in administrative or analytic work, whereas onsite roles may involve clinical or operational responsibilities. As such, some unmeasured job characteristics in this study — such as emotional or physical demands — may contribute to differences in well-being or turnover beyond location of work alone. Additionally, while our well-being measure demonstrated strong reliability and validity, the use of self-reported indicators introduces the possibility of response bias. Nonetheless, these limitations do not undermine the overall pattern of findings but instead highlight useful directions for future research incorporating job-type controls and complementary objective indicators of strain (e.g., absenteeism, health claims).

### Future research

Future research should continue to examine how remote work arrangements influence psychological and relational outcomes over time, particularly through the lens of longitudinal or experimental designs. One promising avenue is to investigate how the quality and source of autonomy—whether self-directed, manager-supported, or structurally embedded—shape well-being and organizational commitment in remote and hybrid settings. Additionally, further exploration is needed to disentangle the mechanisms behind perceived connection in distributed work environments. For example, future studies could assess how different modes of virtual interaction (e.g., synchronous video calls, asynchronous messaging, digital collaboration tools) influence social cohesion and team dynamics.

While our study did not find tenure, age or gender to influence the pattern of relationships observed, research should consider other moderating variables such as personality traits, caregiving responsibilities, and digital fluency, which may affect how individuals experience autonomy and connection across work settings. Future research should seek to replicate these findings in comparable organizational contexts, such as other hospital or healthcare systems, to assess the consistency and generalizability of the observed effects. Replication studies may be particularly valuable for further examining the non-significant findings and determining whether these results reflect contextual factors, sample-specific characteristics, or the absence of meaningful relationships. To that end, cross-cultural or cross-industry comparisons could offer deeper insights into the contextual factors that shape employee experiences in the evolving landscape of work.

### Theoretical implications

These findings also contribute to and extend the dual pathway model proposed by Gajendran et al. (2024), which identifies two competing mechanisms—perceived autonomy and perceived isolation—through which remote work shapes employee outcomes. Our results offer empirical support for the autonomy pathway, demonstrating that digital work arrangements can enhance employees’ control over where, when, and how work is conducted, thereby supporting well-being, focus, and work-life balance. At the same time, contrary to assumptions embedded in the isolation pathway, our data suggest that virtual communication may not uniformly diminish access to support or connection. Instead, in contexts where digital norms are well-established and employee agency is high, remote work may preserve or even strengthen interpersonal connection. This challenges the view that electronically mediated communication is an inadequate substitute for the spontaneous social interactions of the physical workplace. Rather, our findings indicate that digital interactions—potentially shaped by intentional practices, strong communication cultures, and organizational support—may offset or even reverse the isolating effects traditionally associated with remote work. As such, the dual pathway model may benefit from incorporating a more nuanced understanding of how context-specific digital practices and employee agency moderate the balance between autonomy and isolation.

This reinterpretation of the isolation pathway also has implications for how we conceptualize the communicative capacity of digital media, prompting a reexamination of existing frameworks such as media richness theory. That is, the findings call for evolving the framework of media richness theory by suggesting that digital communication tools may now offer sufficient social and informational depth to foster positive interpersonal outcomes, such as connection. Classic media richness theory argues that richer, face-to-face communication is necessary for managing ambiguity and developing relationships (Daft and Lengel, 1986; Ishii et al., 2019). However, the increased prevalence and sophistication of virtual communication in the post-pandemic workplace may have altered what constitutes “rich” media. Our results support a reformulation of the theory in light of context-specific communication dynamics—especially those shaped by employee agency, communication norms, and digital literacy. As we consider the theoretical implications of remote work, we must re-examine existing theoretical models to ensure they have adapted to how workplaces have evolved.

### Practical implications

**Figure 1:**
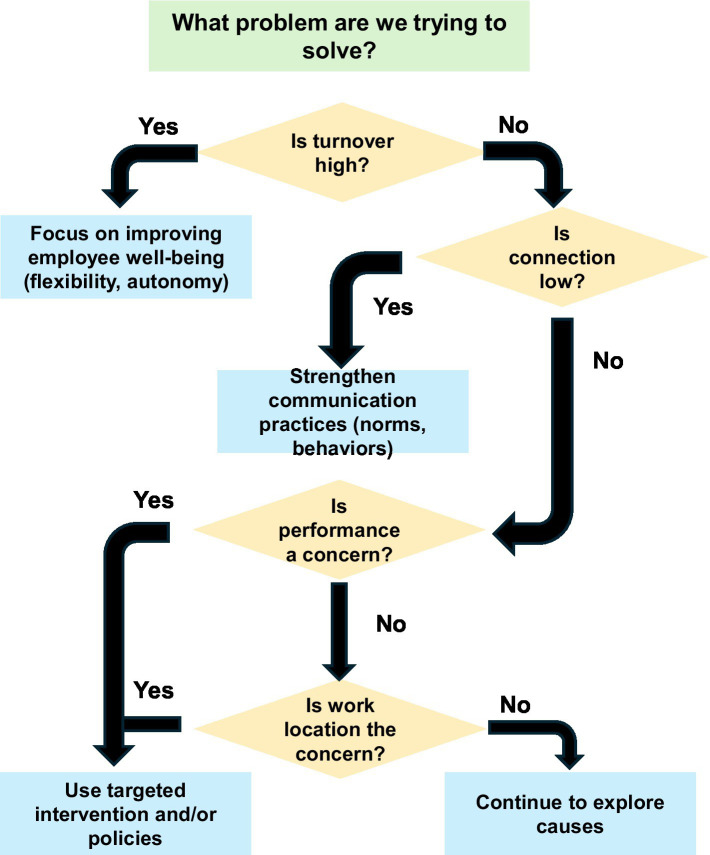
Decision tree for improving connection, well-being, and employee retention.

The implications of this research are especially salient as organizations navigate continued ambiguity in remote work policy. At a time when many organizations are actively debating whether to mandate a return to the office, these findings offer timely and evidence-based insights that challenge prevailing assumptions. Our findings suggest that maintaining or increasing access to remote work opportunities may be a viable strategy for promoting employee well-being—a key determinant of retention. Given the statistically significant relationship between well-being and turnover in our findings, organizations may wish to view well-being not just as an individual concern but as a strategic priority linked to broader organizational stability. Rather than reverting to fully onsite models, leaders should consider a more nuanced approach that centers on flexibility, employee agency, and clearly defining the problem to be solved (Figure 1). The real-world relevance of this study is a significant strength, as its insights directly inform ongoing policy decisions that have substantial implications for workforce satisfaction, equity, and retention.

The results of the current study indicate that many return-to-office decisions are based on assumptions that do not hold under closer examination. Managers can translate these insights into evidence-based management practices that can impact connection, well-being, and employee retention (Table 9). Furthermore, team development exercises can intentionally build behaviors and interaction patterns that foster connection and well-being (for an example, see Table 10).

**Table 9:** From assumptions to evidence-based management.

Common assumption	Evidence-based insight	Implication for managers
Remote work leads to isolation	Connection remains stable, sometimes stronger	Focus on communication design, not physical presence
Employees need to be onsite to stay engaged	Well-being is more strongly tied to engagement and retention	Prioritize flexibility and employee experience
Return-to-office improves retention	Retention is more closely linked to well-being	Avoid mandates that reduce autonomy
Culture depends on proximity	Culture is reinforced through intentional practices	Invest in norms, leadership, and digital collaboration

**Table 10:** Checklist for establishing norms with remote/hybrid employees.

Step	Checklist item	What to do/content
1. Build Understanding (LEARN)	Understand what team norms are	Emphasize that norms are shared expectations for how the team behaves and works together.
	Clarify why norms matter for remote/hybrid teams	Discuss how norms reduce ambiguity, strengthen connection, and support efficient collaboration.
	Review examples	Use sample norms to help the team visualize what norms look like (e.g., direct and respectful communication, learning from mistakes).
2. Create norms (DESIGN)	Ask connection-focused questions	Explore how the team wants to stay connected and handle conflict when not co-located.
	Draft clear, actionable norms	Write norms that describe specific behaviors (e.g., response expectations, meeting etiquette, check-ins).
	Ensure norms support connection	Include behaviors that help people feel included, informed, and supported regardless of physical location.
3. Finalize and Commit (APPLY)	Share norms with team	Send norms to the team for review and adjustments.
	Confirm commitment	Have each team member sign or acknowledge the final set of norms.
	Make norms visible	Post norms in a shared digital space (e.g., Teams channel, OneDrive) and/or include them in meeting openers.
4. Keep Norms Alive (SUSTAIN)	Revisit norms regularly	Review norms during recurring meetings and reflect on how well the team is enacting them. Introduce to new employees as they onboard.
	Adjust as needed	Update norms as roles, work arrangements, or team needs evolve.
	Use norms to guide decisions	Refer back to norms when conflict or misalignment arises.

Team Norms establish clear, agreed-upon behavior, determine how the work will get done, and what team members can expect of each other. They are behaviors that are considered normal, or typical within that group, and are considered a set of shared, informal standards around a topic (e.g., communication, conflict, well-being).

In addition, these results suggest that organizations may not need to sacrifice connection in the pursuit of flexible work arrangements. Perceptions of positive connection were not diminished—and in fact slightly increased—in remote contexts, possibly due to the intentional use of communication tools and rituals that reinforce team cohesion. Organizations should invest in remote communication infrastructures and train leaders in virtual management strategies that foster meaningful interaction. These practices could serve as scalable solutions for preserving culture and morale across distributed teams.

## Conclusion

This study challenges the prevailing narrative that remote work inherently undermines employee well-being and connection. Drawing on a large organizational dataset, we find that remote employees report higher levels of well-being and similar—if not slightly greater—feelings of connection than their onsite counterparts. These findings persist even after accounting for age, gender and tenure. While the magnitude of effects is modest, they do suggest that remote work does not erode, and may even enhance, employee experiences. In an era where debates around flexible work are often shaped more by assumption than evidence, our results offer a data-driven counterpoint to return-to-office mandates predicated on concerns about isolation and disengagement. Ultimately, this research contributes to a growing body of scholarship that advocates for flexibility not as a concession, but as a catalyst for healthier, more connected, and more sustainable work.

## Data Availability

The data supporting the findings of this study are not publicly available due to privacy and ethical restrictions. However, deidentified data may be made available by the corresponding author upon reasonable request, provided that such requests comply with applicable institutional and ethical guidelines.
